# 680. Month Long Fungemia due to *Candida auris* Endocarditis

**DOI:** 10.1093/ofid/ofab466.877

**Published:** 2021-12-04

**Authors:** Haseeba khan, Christy Varughese, Hemil Gonzalez

**Affiliations:** Rush University Medical Center, Chicago, Illinois

## Abstract

**Background:**

*Candida auris (C. auris*) is a multidrug resistant *Candida* species, reported to cause persistent fungemia along with a multitude of invasive fungal infections. We report the first case of *C. auris* fungemia due to endocarditis.

**Methods:**

61 year old man with a history of diverticulitis that required sigmoid resection and was complicated by abdominal abscesses due to multi drug resistant organisms warranting heavy antibiosis. Prolonged hospitalisation for that surgery was followed by a stay at a long term acute care hospital. He was readmitted at an outside hospital with sepsis where blood cultures grew *C.auris*. Upon evaluation, was found to have aortic valve endocarditis. Per patient’s preference, surgery was initially deferred. Despite escalation of therapy with a combination of antifungals, he remained fungemic for five weeks with repeat blood cultures showing changing antifungal susceptibility patterns. Patient eventually underwent surgical intervention at our facility, with valve cultures being positive for *C.auris*. After the surgery he was treated with 6 weeks of intravenous combination antifungal therapy.

**Results:**

*C.auris’s* pathogenicity stems from multiple mechanisms with multi drug resistance being most pertinent. What adds to the complexity of the management is the absence of *C.auris* specific minimum inhibitory concentration breakpoints. Therefore treatment is based on Center for Disease Control’s (CDC) proposed breakpoints that have been extrapolated from other *Candida* spp. It is further complicated by lack of *C.auris* specific data showing essential agreement among different commercially available antifungal susceptibility testing (AFST). Heteroresistance of the microbial population is an issue that must be considered in such protracted fungemia.

**Conclusion:**

Invasive infections due to *Candida auris* presents as a diagnostic and therapeutic challenge to clinicians.

**Disclosures:**

**All Authors**: No reported disclosures

